# Indirect Rectus Femoris Injury Mechanisms in Professional Soccer Players: Video Analysis and Magnetic Resonance Imaging Findings

**DOI:** 10.1097/JSM.0000000000001131

**Published:** 2023-02-28

**Authors:** Aleksi Jokela, Sandra Mechó, Giulio Pasta, Pavel Pleshkov, Alvaro García-Romero-Pérez, Stefano Mazzoni, Jussi Kosola, Filippo Vittadini, Javier Yanguas, Ricard Pruna, Xavier Valle, Lasse Lempainen

**Affiliations:** * Faculty of Medicine, University of Turku, Turku, Finland;; † Department of Orthopaedics and Traumatology, Turku University Hospital, Turku, Finland;; ‡ FC Barcelona, Medical Department, Barcelona, Spain;; § Medical Department, Parma Calcio, Parma, Italy;; ¶ FC Zenit, St. Petersburg, Russia;; ‖ Watford FC, Injury Prevention and Rehabilitation Department, Watford, England;; ** Physiotherapy Department, Universidad Camilo José Cela, Madrid, Spain;; †† Football Club AC Milan, Milan, Italy;; ‡‡ Department of Physical Activity and Health, Paavo Nurmi Centre, University of Turku, Turku, Finland;; §§ Department of Orthopaedics and Traumatology, Kanta-Häme Central Hospital, Hämeenlinna, Finland;; ¶¶ Venezia Football Club, Venice, Italy;; ‖‖ FinnOrthopaedics/Hospital Pihlajalinna, Turku, Finland; and; *** Ripoll y De Prado, FIFA Medical Centre of Excellence, Madrid, Spain.

**Keywords:** rectus femoris, muscle injuries, injury mechanism, video analysis, magnetic resonance imaging, single tendon

## Abstract

**Objective:**

To describe injury mechanisms and magnetic resonance imaging (MRI) findings in acute rectus femoris (RF) injuries of soccer players using a systematic video analysis.

**Design:**

Descriptive case series study of consecutive RF injuries from November 2017 to July 2022.

**Setting:**

Two specialized sports medicine hospitals.

**Participants:**

Professional male soccer players aged between 18 and 40 years, referred for injury assessment within 7 days after a RF injury, with an available video footage of the injury and a positive finding on an MRI.

**Independent Variables:**

Rectus femoris injury mechanisms (specific scoring based on standardized models) in relation to RF muscle injury MRI findings.

**Main Outcome Measures:**

Rectus femoris injury mechanism (playing situation, player/opponent behavior, movement, and biomechanics), location of injury in MRI.

**Results:**

Twenty videos of RF injuries in 19 professional male soccer players were analyzed. Three different injury mechanisms were seen: kicking (80%), sprinting (10%), and change of direction (10%). Isolated single-tendon injuries were found in 60% of the injuries. Of the kicking injuries, 62.5% included complete tendon ruptures, whereas both running injuries and none of the change of direction injuries were complete ruptures. The direct tendon was involved in 33% of the isolated injuries, and the common tendon was affected in all combined injuries.

**Conclusions:**

Rectus femoris injuries typically occur during kicking among football players. Most of the RF injuries involve a complete rupture of at least one tendon. Kicking injuries can also affect the supporting leg, and sprinting can cause a complete tendon rupture, whereas change of direction seems not to lead to complete ruptures.

## Abstract 2

Supplemental Digital Content is Available in the Text.

## INTRODUCTION

Quadriceps muscle injuries are common in sports requiring sprinting and kicking.^1–5^ In football, thigh muscle injuries are the most common injuries, rectus femoris (RF) being the most frequent location in the quadriceps^4,6–9^

When deciding the best treatment for a muscle injury, a correct diagnosis and injury classification are the first steps.^10^ A full comprehension of the muscle belly affected, the injury location, and an evaluation of the connective tissue damage are the basis for designing the treatment.^10,11^ The most common treatment approach for RF injuries is nonoperative, but there are certain indications in which surgical treatment should be considered.^5,12–15^

Understanding newly discovered anatomical structures of the RF can aid in diagnosis and treatment. It is well known that the proximal myotendinous junction (MTJ) has 2 origins, the direct and the indirect head.^16^ The direct head arises from the anterior–inferior iliac spine, and the indirect head arises from the superior acetabular ridge and the posterolateral aspect of the hip joint capsule.^16^ The 2 tendons blend and form a conjoined tendon (CT) about 2 cm distal to their origin, with the direct head located at the superficial part of the CT.^16^ Each tendon maintains a separate structure, with 10% to 20% of fibers decussating.^16^ After the CT, the direct head extends distally on the surface of the muscle for one-third of the belly's length, and the indirect portion becomes intramuscular and extends about two-thirds of the muscle length. The distal MTJ extends for the distal two-thirds of the muscle belly length, and it is located in the dorsal surface of the belly.^16^

When it comes to the lesser-known anatomy of the RF, it has been recently shown that there is a third membranous origin, which connects the CT to the anterior–superior iliac spine.^17^ In addition, the existence of a third head of the RF extending from the inferior edge of the indirect tendon to the greater trochanter blending with the gluteus minimus has been described by Tubbs et al.^18^ This anatomical variant was present in 83% of the thighs dissected in their study. These 2 newly discovered structures, the third membranous origin and the third head of the RF, should be considered in future algorithms used to diagnose RF injuries.

Indirect injury mechanisms may be multifactorial, but kicking and sprinting have been described as the main actions involved in RF injuries.^19,20^ Kicking has been found to be an important mechanism related to complete tears and injuries occurring at the proximal free tendon.^20^ Understanding the injury mechanisms and injury-inciting events is crucial to develop prevention methods for sports injuries.^21^ In describing injury mechanisms, athlete interviews are a simple way to reach large sample sizes, but they also present limitations; these include the lack of a precise definition of the injury moments and the lack of ability of the injured players to comprehend and recall reliably what actually happened when the injury occurred.^22^ Video footage of injury, with frame-by-frame slow motion and video stoppage, can provide a precise description of the factors leading to an injury, and the value of such video analysis has been demonstrated for other sports injuries.^23–26^

Although the main mechanisms of RF injuries have been described in the literature, no previous video analysis studies, combined with magnetic resonance imaging (MRI) findings, have been published to our knowledge. There is a need for accurate information about the RF injury mechanisms and patterns, so that more efficient injury prevention, diagnostics, and management can be developed. The main aim of this study was to describe the mechanisms, situational patterns, biomechanics, and MRI findings related to RF injuries in professional male soccer players using a systematic video analysis.

## METHODS

### Subjects

Professional male soccer players with an acute RF injury were consecutively included from 2 private departments at specialized sports medicine hospitals in Finland and Spain from November 2017 to July 2022. The inclusion criteria were as follows: professional male soccer player (aged 18-40 years), with initial and acute onset anterior thigh pain occurred while training or competing. In addition, subjects had to have a confirmed RF injury on an MRI that was performed within 7 days of the date of injury as well as video footage of the injury when it occurred. The exclusion criteria were pain of nonmusculotendinous cause, inadequate quality of video footage, or refusal to allow the use of video footage.

### Video Acquisition and Processing

All included RF injuries were broadcasted on television or recorded by a training crew while filming performance according to standard protocol. TV-broadcasted video footage was accessed through the teams' archiving system or through public sources. The videos were stored in an MP4 format. Injury sequences were cut, processed, and edited using Final Cut Pro V.10.5.2 and Wondershare Filmora9 V.9.5.3 software with all files converted to QuickTime (.mov), allowing frame-by-frame navigation using QuickTime player V.10.4. In a further stage in the video processing, we followed the steps presented in a study published by Serner et al.^26^ To obtain a good demonstration of the injury situation, the video was cut from the start of the performance before the injury to a stop of the performance immediately after the injury. In addition, shorter clips were made, which included footage of the specific injury mechanism from each available camera view. This means that there was one clip of the full situation, as well as 1 to 4 additional slow motioned clips depending on the number of available camera angles, allowing easy frame-by-frame navigation.

### Determination of Injury Movement

Each injury situation was reviewed and discussed with each injured athlete to determine the specific movement and body position in which the player recalled feeling the pain. This review was performed within 24 to 48 hours of the initial injury in most cases. In addition, the assumed time of injury was estimated by 3 authors (A.J., X.V., and L.L.) based on the injury mechanism, body positions, and athlete reactions. Based on this information, the assumed exact injury frame was defined.

### Video Analysis

The athletes' own narrative descriptions were reported focusing on the events leading to the injury, the actual moment of pain sensation, and their capability to continue playing/training/performing.

Based on a comprehensive model for injury causation^21^ and standardized scoring forms,^23–26^ a specific RF questionnaire was developed to describe accurately the injury mechanism and the events leading up to the injury. Two authors (A.J. and X.V.) were asked to complete the standardized form involving specific questions about the playing situation, player/opponent behavior, movement, and biomechanical body positions at the defined injury moment (see **File 1**, **Supplemental Digital Content 1**, http://links.lww.com/JSM/A364). The analysts (A.J. and X.V.) scored the videos independently. Any discrepancies in the scoring were noted and discussed in a consensus meeting, where videos were critically viewed again until a consensus was reached. Scoring was performed and analyzed using Excel 2018 (Version 16.16.27).

### Muscle Magnetic Resonance Imaging Exams

All the explorations were performed by 3T MR scans, in 2 different radiology departments, on a 3T Canon Vantage Titan (Canon Medical Systems, Japan) in the FCB Magnetic Resonance Center and on a Siemens Skyra 3T MRI Scanner in the Radiology Unit of a private sports hospital in Turku, Finland. The muscle MR protocol included axial, sagittal, and coronal fat-suppressed T2-weighted sequences and axial and coronal T1-weighted sequences. All the studies included the pelvis and both thighs.

### Magnetic Resonance Imaging Analysis

Two experienced radiologists independently assessed all the MR images to evaluate possible lesions of the RF. In the diagnosis, accurate interpretation was based on the general muscle lesion patterns already described in the literature^27–30^ and on the extension and location of the connective tissue damage. In the cases of proximal injury, the analysis method consisted of studying the proximal MTJ complex of the RF and its distal extension of the CT, and more distally the direct portion and the central tendon (also known as the septum). In the cases with distal injury, the analysis method consisted of studying the distal MTJ of the RF following its proximal extension. Tendinous tear was identified as a hyperintense gap on T2 with a loss of tendon continuity, which could be partial or complete depending on the tendinous cross-sectional area injured. The location and amount of connective tissue damage, the presence or absence of edema, and the characteristics of edema were used to evaluate the severity of injury.^31^

## ETHICAL CONSIDERATIONS

The study protocol was reviewed by the Ethics Committee of the Hospital District of Southwest Finland (ETMK 111/1801/2020). The assessment of the ethics committee was that according to Finnish law no ethical review by the regional ethics committee (Act 499/1999) was necessary or required beforehand. All included patients were informed about the study setup, they participated on a voluntary basis, and consent was acquired from all athletes at inclusion according to the Declaration of Helsinki.

## RESULTS

### Subjects

Twenty videos of acute RF injuries of 19 professional male soccer players met the inclusion criteria. One of the included athletes had a recurrence of the injury 8 weeks after the initial injury. Therefore, 20 cases were included (median age: 24.5 years, range 18-38 years). Of the 19 athletes, there were 4 goalkeepers and 15 outfield players. The characteristics of each case are presented in Table 1.

**TABLE 1.** Characteristics of Each Case

Case	Action at Injury	Concurrent Movement	If Kicking: Type/Ball Impact/Ball Moving/Kicking Leg Injured	If Running: Speed	If Changing Direction: Angle/Direction	Balance	Contact: Type/When	MRI Finding (Complete Ruptures Bolded)
Kicking								
**1**	Kicking	Running	Cross/side foot/yes/yes	High	—	No	—	IT + **CT**
**2**	Kicking	Running	Shot on goal/side foot/yes/yes	High	—	Yes	—	**DT**
**3**	Kicking	Jogging	Shot on goal/side foot/yes/yes	Low	—	Yes	—	Distal MTJ
**4**	Kicking	Jogging	Shot on goal/instep/yes/yes	Low	—	Yes	—	IT + CT + **DDP**
**5**	Kicking	Jogging	Clearing/instep/no/yes	Low	—	Yes	—	**CT + DDP + CS**
**6**	Kicking	Jogging	Shot on goal/instep/yes/no	Low	—	No	—	CS
**7**	Kicking	Jogging	Goal kick/instep/no/yes	Moderate	—	Yes	—	**DT**
**8**	Kicking	Running	Shot on goal/instep/yes/no	High	—	No	—	**Distal MTJ**
**9**	Kicking	Jogging	Cross/instep/yes/yes	Low	—	Yes	—	CT
**10**	Kicking	Jogging	Goal kick/instep/no/yes	Low	—	Yes	—	**CT**
**11**	Kicking	Jogging	Long pass/side foot/yes/yes	Low	—	Yes	—	DT + IT + CT
**12**	Kicking	Walking	Clearing/instep/yes/yes	Low	—	Yes	—	DT
**13**	Kicking	Jogging	Goal kick/instep/no/yes	Low	—	Yes	—	**DT**
**14**	Kicking	Maximal sprinting	Clearing/toe kick/yes/yes	Very high	—	Yes	—	**Distal MTJ**
**15**	Kicking	Running	Long pass/side foot/yes/yes	High	—	Yes	—	**IT + CT**
**16**	Kicking	Running	Shot on goal/Side foot/yes/yes	High	—	Yes	—	IT + CT + DDP
Running								
**17**	Running (in speed)	Maximal sprinting	—	Very high	—	Yes	Indirect, shoulder/before the injury	IT + **CT**
**18**	Running (in speed)	Maximal sprinting	—	Very high	—	No	—	**CT**
Change of direction								
**19**	Change of direction	Standing	—	High	<45 degrees/toward injured side	No	—	CS
**20**	Change of direction	Running	—	High	>90 degrees/toward uninjured side	No	Indirect, shoulder/before the injury	CT + DDP

CS, central septum; DT, direct tendon; DDP, distal direct portion; IT, indirect tendon.

### Injury Mechanisms

**Figure 1.**
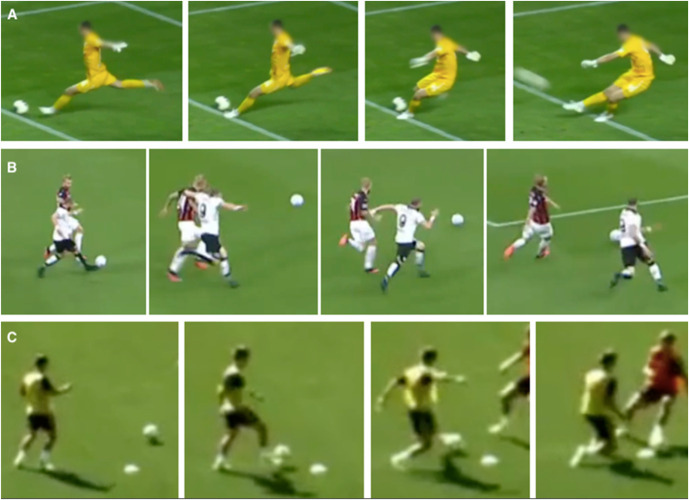
A: kicking, B: sprinting, C: change of direction.

Three different injury mechanisms were seen in the video analysis: kicking (80%), sprinting (10%), and change of direction (10%). The examples of the injury mechanisms can be found in Figure 1. Video examples of all 3 mechanisms are presented in see **File 1**, **Supplemental Digital Content 2 to 4** (see **Files 2 to 4**, http://links.lww.com/JSM/A365, http://links.lww.com/JSM/A366, and http://links.lww.com/JSM/A367). In kicking injuries, the type of kick varied, and the ball impact occurred at the instep in 56% of the cases, whereas in 38% of the cases, the player kicked the ball with his side foot. In 2 cases, the player injured his supporting leg while executing a kick involving a rapid change of movement from hip flexion to extension and knee extension to flexion. The rest of the kicking injuries involved a rapid change of movement from hip extension to flexion and knee flexion to extension. Both sprinting-type injuries occurred during maximal sprinting at a very high speed. Both change of direction injuries occurred during the handling of the ball, one involved an angle of <45 degrees toward the injured side, and the other an angle of >90 degrees toward the uninjured side. All players were able to determine the situation and movement causing the pain in the RF area. Descriptive information on the biomechanics in each case are presented in Table 2.

**TABLE 2.** Biomechanical Characteristics of the Cases

Injury Action	Rapid Change of Movement Involving Hip Extension to Hip Flexion	Rapid Change of Movement Involving Hip Flexion to Hip Extension	Rapid Change of Movement Involving Knee Flexion to Knee Extension	Rapid Change of Movement Involving Knee Extension to Knee Flexion	Open Chain	Closed Chain
Kicking (with injured leg)	14/14	0/14	14/14	0/14	14/14	0/14
Kicking (with uninjured leg)	0/2	2/2	0/2	2/2	0/2	2/2
Running	N/A	N/A	N/A	N/A	N/A	N/A
Change of direction	0/2	1/2	0/2	1/2	0/2	2/2

N/A, not available.

### Magnetic Resonance Imaging Findings

**Figure 2.**
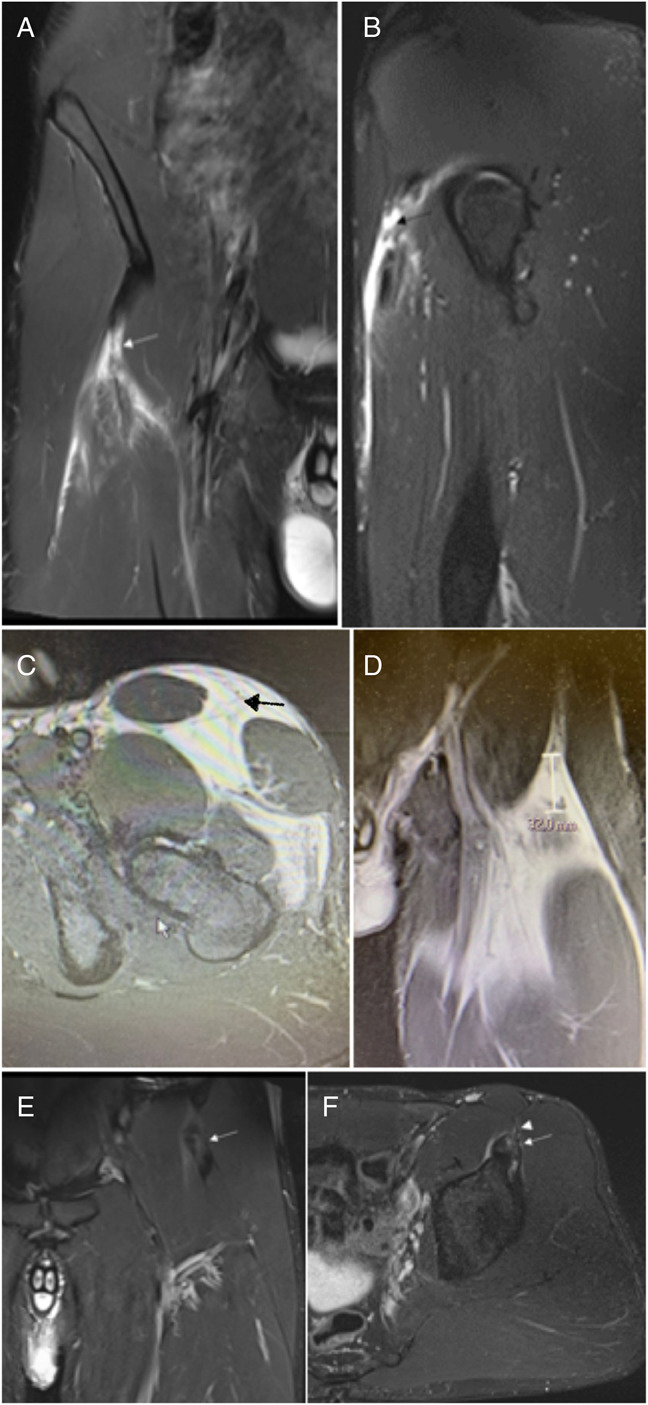
A: Coronal fat saturated proton density image that shows a complete rupture of the right RF common tendon. There is a gap of connective tissue (arrow), slight lost of tendinous tension, and loss of pennation angle. B: Sagittal fat saturated proton density image that shows the complete rupture of the common tendon (arrow). C: Axial fat saturated proton density image that shows the absence of left RF due to a complete rupture of the proximal tendinous complex (arrow). D: Coronal fat saturated proton density image where there is a distal retraction of the left RF of 3 cm. E and F: Coronal (E) and axial (F) fat saturated proton density images that show a hypertrophic and heterogeneous callus of the left RF direct tendon (arrow). The membrane that communicates the common tendon with the anterosuperior iliac spine is also thick with scar changes (arrowhead).

**Figure 3.**
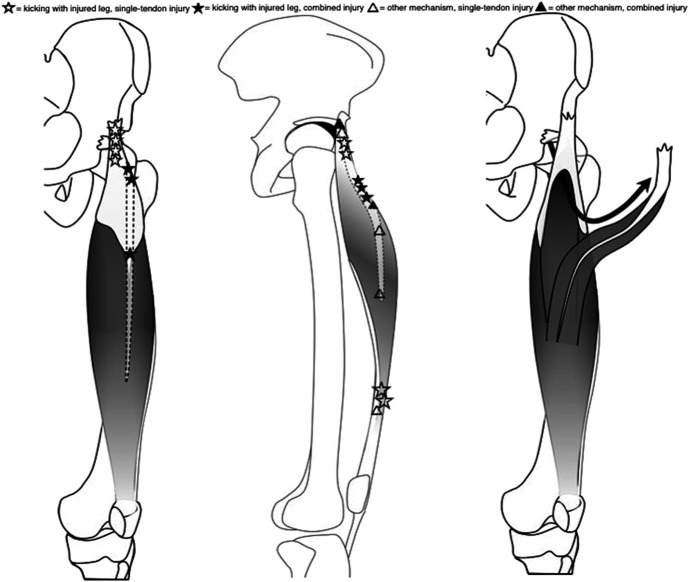
Locations of injuries and their relation to injury mechanism and injury type.

Findings of MRI with the corresponding injury mechanisms are presented in Table 3. In total, 12 injuries were isolated single-tendon injuries (4 DT, 3 CT, 2 CS, 3 distal MTJ), whereas 8 cases involved combined injuries. In 12 cases, the rupture was classified as complete and as partial in 8 cases. The examples of the MRI findings are presented in Figure 2. The illustration of the locations of injuries and their relation to injury mechanism and injury type is presented in Figure 3.

**TABLE 3.** Injury Mechanisms and MRI Findings

MRI Finding	Kicking	Running	Change of Direction	Total
Isolated injuries	10	1	1	12
DT	4	—	—	4
IT	—	—	—	—
CT	2	1	—	3
DDP	—	—	—	—
CS	1*	—	1	2
Distal MTJ	3†	—	—	3
Combined injuries	6	1	1	8
IT + CT	2	1	—	3
IT + CT + DDP	2	—	—	2
CT + DDP + CS	1	—	—	1
CT + DDP	—	—	1	1
DT + IT + CT	1	—	—	1
Complete‡/partial	10/6	2/0	0/2	12/8

* The supporting leg was injured in this case.

† The supporting leg was injured in one of the cases.

‡ The combined injury was classified as complete, if it included at least one complete tendon rupture.

CS, central septum; DT, direct tendon; DDP, distal direct portion; IT, indirect tendon.

## DISCUSSION

Our findings confirm the knowledge that RF injuries usually occur during activity requiring eccentric muscle action, such as kicking and sprinting. We demonstrated that systematic video analysis is very accurate way to evaluate RF injury mechanisms, which helps to deepen the understanding of the multifactorial etiology of these injuries. The injury was most commonly located in the common tendon of the proximal RF MTJ, but injuries to the distal MTJ were also found. To our knowledge, this is the first study investigating RF injury mechanisms in detail by using video analysis combined with MRI findings.

Based on the findings of this study, we suggest that video analysis can be an excellent tool when assessing acute RF injuries, understanding the details of the injury mechanisms, developing preventive strategies, and remote consultation between the clinicians.

### Kicking Injury Mechanisms

There is no clear evidence as regards the details of RF injury mechanisms because previous studies using accurate methods to investigate injury patterns are lacking. In this study, kicking was the most common injury mechanism, which is in contrast with previous studies concerning the association between an RF injury and the injury mechanism.^6,20^ Geiss Santos et al^20^ recently found that there is an association between a kicking injury mechanism and complete RF ruptures and an injury to the proximal free tendon. In the study performed by Geiss Santos et al,^20^ the injury mechanism was classified as kicking if the injury occurred during a kicking action and no detailed analysis or objective confirmation of the injury mechanism was used. In other previous studies concerning MRI-confirmed RF injuries, the injury mechanism has not been detailed.^1,3,7^

To understand the details of RF injury mechanisms, it is essential to be aware of the basics of biomechanics during kicking. A kicking motion begins with the back-swing phase (early swing phase) during which the RF works eccentrically as it acts to decelerate the hip extension and knee flexion.^32,33^ During the early part of the forward-swing phase (wind-up phase), the hip starts to flex, whereas the knee is still flexing while the RF is contracted eccentrically to prevent excessive knee flexion.^34^ Subsequently, the angular velocity starts to increase in both the thigh and lower leg until the angular velocity of the thigh reaches its maximum at 75% of the total swing time.^32^ Lower leg angular velocity keeps increasing until the ball contact, during which the RF is in a relatively shortened state and the knee is extended.^32^ Mendiguchia et al^19^ suggested that during the wind-up phase, the RF is prone to injury due to the greater angular velocities and greater knee flexion.

The most frequently used soccer kicks are the full-instep kick (“full kick”) and the inside-of-the-foot passing shot (“pass kick”). In the full kick, the ball is hit by the medial–superior part of the instep, and it is normally used to generate a fast ball speed.^33^ In the pass kick, instead, the ball is hit by the medial portion of the foot, and the main priority is to kick the ball more precisely.^33^ Levanon et al showed that in the pass kick, the thigh remains in an externally rotated orientation throughout the kicking motion, whereas in the full kick, the thigh is initially in an externally rotated orientation and subsequently rotates counterclockwise facing almost directly forward until the ball impact. However, in both the full kick and pass kick, most of the speed of the foot during the ball impact is still generated through the knee's extension.^33^ In our sample, 56% of the kicking injuries occurred during a full kick, 38% during a pass kick, and 6% during a toe kick.

Ten of the kicking injuries included complete ruptures of at least one tendon. Ten injuries were located in the proximal free tendon, 3 in the distal MTJ, and 3 in the proximal MTJ and/or intramuscular tendon. These findings are in line with the study of Geiss Santos et al, in which the findings showed kicking as being related to complete ruptures and injuries occurring in the proximal free tendon.^20^ In this study, 2 of the 16 kicking injuries affected the supporting leg, which is an example of the accuracy of the video analysis as Geiss Santos et al did not report whether the RF injuries during kicking affected the kicking or supporting leg. We suggest that the athlete's own recollection or the medical staff report based on eyewitnesses are not as accurate ways to evaluate injury mechanisms as video analysis. If video footage of an injury is available, it should always be used because it offers objective and detailed evidence of the injury mechanism, which makes the diagnosis, rehabilitation, and prevention easier.

### Nonkicking Injury Mechanisms

In this study, 4 injuries occurred during sprinting or a change of direction. Mendiguchia et al^19^ described that RF injuries can occur during sprinting, especially during acceleration or deceleration. They suggested that high angular velocities of the hip and knee during the swing phase combined with high eccentric activity make the RF more prone to injury. During sprinting, the maximum length of the RF occurs during the swing phase and takes place at approximately 55% of the sprint cycle, between the peak hip extension (40%) and the maximal knee and hip flexion (65%).^35^ Conversely, soccer players often make sudden movements by changing direction, decelerating, or stopping rapidly. In these actions, the breaking forces from deceleration create high eccentric load and must be rapidly absorbed throughout the muscles in the lower limb. This can lead to injury.^36^

### Clinical Aspects

Based on the authors' clinical experience, soccer players with RF injuries are often able to run, jump, sprint, and make rapid changes of direction without any discomfort after 4 weeks of rehabilitation, but kicking the ball is still painful and very easily leads to reinjuries. Therefore, the main concern with a soccer player suffering from the RF injury is the ball contact, mainly kicking and passing. Kicking the ball in soccer is an action requiring great explosive forces with much of the work performed eccentrically making it logically an injury prone activity. In addition to primary RF injuries, reinjuries are also likely to happen when kicking the ball. Thus, in the case of acute RF injury, careful rehabilitation planning and an awareness of the reinjury risk factors are highly recommended.

Video analysis of injury mechanisms can reveal individual risk factors, such as injury-prone technique during running or kicking, which can be taken into consideration when developing preventive strategies for RF injuries. Video analysis is also a helpful tool in diagnostics because kicking more often leads to complete RF ruptures and injuries to the free tendon. However, we showed that sprinting-type RF injuries can also lead to complete ruptures of the free tendon. It is essential to know that other more untypical injury mechanisms also occur, and these can as well cause some of the more severe injury patterns. Knowledge about the relationship between injury mechanisms and injury types is crucial when making treatment decisions because the severity and location of the injury is significant when deciding whether the injury is treated surgically or conservatively.

### Limitations

Some obvious limitations can be identified in this study involving the relatively small sample size. However, the injury situations described in this study were consistent with the findings of the previous literature, and the purpose of the study was to provide further and more specific information about RF injury mechanisms causing different injury types using video analysis and MRI findings. In addition, all cases included in this study were indirect muscle strain injuries. Despite being so common in soccer, direct contusion injuries to the anterior thigh were not included in this study because the mechanisms of such injuries are quite straightforward, whereas the RF strain injury mechanisms have not been completely solved, and there is a considerable need the for development of prevention strategies in this field. In addition, one case was a confirmed reinjury, which has to be taken into account in evaluating the correlation between injury mechanism and injury type. Finally, the validity of the defined injury moment was based on the athletes' recollections and slow-motion video footage; nevertheless, it is still difficult to be certain that the injury occurred at the exact described moment. Despite the limitations mentioned, a detailed video analysis of authentic injury situations seems to offer more specific and accurate information about injury mechanisms and provides reliable evidence of the injury patterns. Further research on acute muscle injury mechanisms using video analysis is suggested.

## CONCLUSIONS

In this study, we present the detailed video analysis regarding different RF injury mechanisms combined with MRI findings. Injury patterns can be categorized into kicking, sprinting, and change of direction, during which indirect forces can cause severe RF injuries. We found that not only kicking but also sprinting can cause complete proximal tendon ruptures, whereas change of direction caused only partial ruptures. This study highlights the value of video analysis in the everyday clinical practice of sports physician.

## Supplementary Material
